# Enhanced resistance to calcium poisoning on Zr-modified Cu/ZSM-5 catalysts for the selective catalytic reduction of NO with NH_3_

**DOI:** 10.1039/c9ra07722g

**Published:** 2019-11-25

**Authors:** Hongyan Xue, Tao Meng, Fangfang Liu, Xiaoming Guo, Shengdong Wang, Dongsen Mao

**Affiliations:** Research Institute of Applied Catalysis, School of Chemical and Environmental Engineering, Shanghai Institute of Technology Shanghai 201418 PR China guoxiaoming@sit.edu.cn dsmao@sit.edu.cn +86 21 60873301 +86 21 60873301; Shanghai Institute for Design & Research in Environmental Engineering Co., Ltd. Shanghai 200232 PR China

## Abstract

Ca/ZrCu/ZSM-5 catalysts containing different Zr contents were prepared by incipient wetness impregnation. The catalysts were tested for the selective catalytic reduction (SCR) of NO_*x*_ with ammonia and characterized by N_2_-BET, N_2_O titration, XRD, NH_3_-TPD, H_2_-TPR, and XPS techniques. In the temperature range of 100–170 °C, after calcium impregnation, NO_*x*_ conversion over the Cu/ZSM-5 catalyst decreased by 11.3–24.3%, while that over Zr_0.10_/Cu/ZSM-5 only decreased by 3.8–12.2%. The improvement of the calcium poisoning resistance of the ZrCu/ZSM-5 catalyst is mainly attributed to an increase in the dispersion and the surface concentration of Cu. Moreover, the addition of zirconium promotes the reduction of CuO by decreasing the interaction between CuO and CaO, which also contributes to the improvement of resistance to CaO poisoning. The apparent activation energy and turnover frequency for the SCR reaction over the Ca/Zr_*x*_Cu/ZSM-5 catalysts were calculated and discussed.

## Abstract 2

After calcium impregnation, NO_*x*_ conversion over the Cu/Z catalyst decreased by 11.3–24.3%, while that over Zr_0.10_/Cu/Z only decreased by 3.8–12.2%.

## Graphical abstract

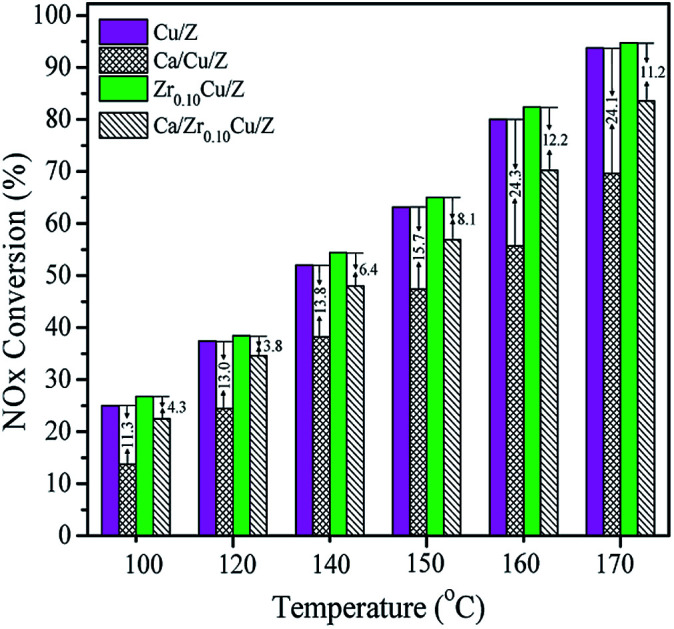

## 1. Introduction

As major air pollutants, nitrogen oxides (NO_*x*_) result in a series of environmental issues, such as photochemical smog, acid rain, and ozone depletion.^1–3^ Recently, researchers found that NO_*x*_ is also one of the key factors for the formation and evolution of haze, which is harmful to the ecological environment and human health.^4^ Many effects have been devoted to reduce the emission of NO_*x*_. Among the technologies for the removal of NO_*x*_, selective catalytic reduction (SCR) of NO_*x*_ with ammonia is the most effective and economical technique.^5–9^

Due to the poor low-temperature catalytic activity of vanadia-based catalysts,^10–12^ the SCR reactor must be installed upstream of the desulfurization system and electrostatic precipitator. Several common poisons in the fly ash including SO_2_ and heavy metals, especially alkali/alkaline earth metals deactivate the catalyst severely.^13,14^ Cu-based catalysts have been deemed to be a competitive candidate for SCR reaction because of its excellent low-temperature activity, remarkable hydrothermal stability and environmentally benign property.^5,15–18^ The SCR reactor using Cu-based catalyst could be placed downstream of the desulfurization system and electrostatic precipitator, and the harmfulness of fly ash to the catalyst was cut down remarkably. However, trace alkali metal and alkaline earth metal still remains in the flue gases, and the poisoning effect is inevitable.^19–21^ Putluru *et al.*^19^ reported that the decrease in the catalytic activity of Cu/zeolite catalyst by K loading was mainly attributed to the loss of the surface acid sites and the decline of the ability for reduction of CuO. Fan *et al.*^20^ pointed out that the NH_3_-SCR activity on the Cu/SSZ-13 catalyst was greatly reduced with the introduction of alkali metal. They indicated that the zeolite structures of the poisoned catalyst were seriously deteriorated and the amount of active sites (Cu(ii)) was decreased significantly. Thus, it is necessary to improve the resistance of catalyst to alkali metals and alkaline earth metals. Many researchers attempt to improve the resistance of vanadium-based and manganese-based catalysts to alkali and alkaline earth metals.^22–26^ For instance, Due-Hansen *et al.*^22^ pointed out that the resistance to potassium poisoning of the vanadia-based catalysts supported on tungstated and sulfated zirconia was significantly improved comparing with the traditional V_2_O_5_/TiO_2_ catalyst. In that case, the potassium preferentially interacted with the sulfate and/or tungstate, and the poisoning effect was alleviated largely. Peng *et al.*^26^ reported that the potassium poisoning of MnO_*x*_/TiO_2_ catalyst is due to the decline of reducibility, the decrease in surface acidity and the enhanced stability of nitrite/nitrate species. After the modification with CeO_2_, the resistance to the potassium poisoning was improved, and the reasons were ascribed to that the cerium could provide the surface acid sites, which was difficult to be neutralized by alkali atoms. Moreover, the influence of potassium on the 4f orbitals of Ce is negligible, which stabilizes the reducibility of catalyst for NH_3_ activation. Unfortunately, the investigations concerning the improvement of calcium resistance of the Cu/zeolite catalysts for the selective catalytic reduction of NO_*x*_ are quite few.

In this study, Ca/ZrCu/ZSM-5 catalysts containing different Zr content were prepared and tested for the SCR of NO_*x*_. This study focuses on the promoting effects of ZrO_2_ on the resistance to Ca poisoning over the Cu/ZSM-5 catalyst. Moreover, the physicochemical properties of the catalysts were investigated, and the relationship between the physicochemical properties and the activity of catalysts was discussed.

## 2. Experimental section

### 2.1. Catalyst preparation

ZrCu/ZSM-5 catalysts containing a constant copper content of 4 wt% and variable amount of zirconium were prepared with the impregnation method.^21^ The commercial zeolite H-ZSM-5 (Si/Al = 38) was purchased from the catalyst plant of Nankai University, Tianjin, China. Typically, a certain amount of aqueous solutions of Cu(NO_3_)_2_·3H_2_O and Zr(NO_3_)_4_·5H_2_O were slowly dropped into the support H-ZSM-5 and stirred at room temperature. The as-impregnated precursor was dried at 120 °C and then calcined at 550 °C for 4 h in air atmosphere. The as-prepared catalysts were termed as Zr_*x*_Cu/Z, where *x* is the weight ratio of Zr to ZSM-5 and expresses in percentage; for example, *x* = 0.05 means that the weight percentage of Zr to ZSM-5 equals 0.05%. The CaO-impregnated Zr_*x*_Cu/Z catalysts, with a constant weight ratio of Ca to ZSM-5 of 1.26%, were prepared *via* impregnating the Zr_*x*_Cu/Z catalysts with Ca(NO_3_)_2_ solution and denoted as Ca/Zr_*x*_Cu/Z. Prior to the evaluation of the catalytic activity, the catalysts were tableted, and then granulated and screened to a size of 40–60 meshes.

### 2.2. Catalyst characterization

Full nitrogen adsorption/desorption isotherms at −196 °C were obtained after degassing at 200 °C, using an adsorption apparatus (Micromeritics ASAP-2020 HD88). The specific surface areas of catalysts (*S*_BET_) and the average pore size were estimated with the BET and BJH method.

The copper surface area (*S*_Cu_) was measured by N_2_O pulse titration using a gas sampling valve. Approximately 0.1 g of sample was packed into a quartz tube reactor and reduced in an H_2_/N_2_ mixture (10 vol%) for 1 h at 300 °C. After being purged with He to remove any weakly adsorbed H_2_, the catalyst was cooled to 80 °C under He. Next, a gas mixture of N_2_O/He (2 vol%) was intermittently injected into the reactor until the reaction was completed. N_2_O and N_2_ in the effluent were analyzed by a mass spectrometer (Pfeiffer Vacuum). The metallic copper surface area was calculated from the following equation assuming an atomic copper surface density of 1.46 × 10^19^ atoms per m^2^.^27–29^

1 2Cu_(s)_ + N_2_O_(g)_ = Cu_2_O_(s)_ + N_2(g)_

X-ray diffraction (XRD) pattern was recorded on a PANalytical X'Pert instrument with Ni-filtered Cu *K*_α_ radiation at 40 kV and 40 mA. Scans were made in the 2*θ* range of 20°–60° with a scanning speed of 6° min^−1^.

Temperature-programmed desorption of ammonia (NH_3_-TPD) was employed to determine the surface acidity of the samples. First, approximately 0.1 g of the sample was flushed with N_2_ gas at 400 °C for 20 min. Then, the sample was cooled to 50 °C and saturated with ammonia at the same temperature. Subsequently, a pure N_2_ stream was passed over the catalysts at 100 °C in order to remove the physisorption molecules. TPD experiments were started with a heating rate of 5 °C min^−1^ from 100 to 600 °C.

H_2_-TPR was performed in a continuous-flow apparatus equipped with a thermal conductivity detector (TCD). First, 50 mg of sample was purged by N_2_ at 400 °C for 20 min. After cooling down to room temperature, the reducing gas of 10% H_2_/N_2_ was switched on, and then H_2_-TPR measurements were carried out with a heating rate of 5 °C min^−1^. The amount of consumed H_2_ was measured by the TCD.

The XPS and Auger electron spectroscopy were recorded on an ESCALA 250 Xi spectrometer using a standard Al *K*_α_ X-ray source (1486.6 eV). The binding energy value was calibrated with C 1s = 284.6 eV as a reference. The reduction of catalyst was performed in a separate reaction chamber, and the samples were transferred by a transfer rod without exposure to air.

### 2.3. Catalytic activity and kinetic tests

SCR activity measurement was carried out in a flow-type apparatus designed for continuous operation at atmospheric pressure. The reactant gas was composed of 500 ppm NO, 500 ppm NH_3_, 5% O_2_ and balanced N_2_. 0.15 g of catalyst was packed into a fixed-bed quartz reactor with an inner diameter of 6 mm. The space velocity was 80 000 ml g^−1^ h^−1^ and the reaction temperature ranged from 100 to 400 °C. The composition of the exhaust gases was monitored by flue gas analyzer (Testo 340), and the conversion of NO_*x*_ was calculated using the following equation:2
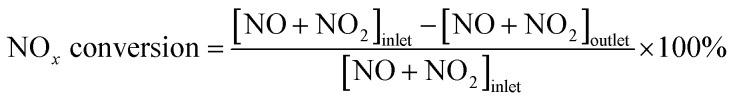


Steady-state reaction rates were obtained from 100 to 150 °C, and the SCR reaction rates per gram of catalyst were calculated by the following equation:3
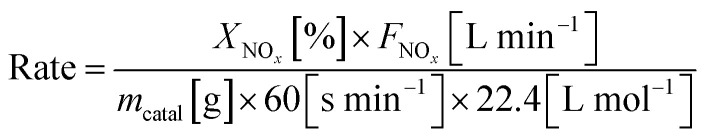


The turnover frequency (TOF) of the NH_3_-SCR reaction, defined as the number of NO_*x*_ molecules converted per metallic copper atom per second, can be calculated by the equation:4
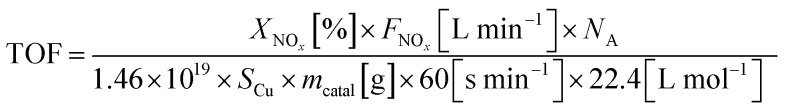
where, *X*_NO_*x*__ is the conversion of NO_*x*_ (eqn (2)), *F*_NO_*x*__ is the volumetric flow rate of NO_*x*_, *m*_catal_ is the mass of catalyst, *N*_A_ is the A Fugadero constant, and *S*_Cu_ is the copper surface area determined by N_2_O titration (eqn (1)).

## 3. Results and discussion

### 3.1. Textural and structural properties

Table 1 shows the textural properties of the investigated catalysts. The BET surface area (*S*_BET_) of the Cu/Z catalyst is 267.3 m^2^ g^−1^, which decreases to 249.1 m^2^ g^−1^ after Ca loading. For the Ca/Zr_*x*_Cu/Z catalysts, with the increase in Zr content, there is a little change in the BET surface area, the total pore volume and the average pore diameter. However, as the zirconium content reached 0.40 wt%, the *S*_BET_ declined to a value of 220.8 m^2^ g^−1^. The reason may be ascribed to the blocking of zeolite pores by CaO and ZrO_2_. For comparison, the textural properties of Zr_0.10_Cu/Z are also presented in Table 1. The *S*_BET_ value (279.1 m^2^ g^−1^) of Zr_0.10_Cu/Z is slightly larger than that of Cu/Z catalyst.

**Table 1:** Physical property of the Cu/Z and Ca/Zr_*x*_Cu/Z catalysts

Catalyst	BET surface area (m^2^ g^−1^)	Total pore volume (cm^3^ g^−1^)	Average pore diameter (nm)	Cu surface area^a^ (m^2^ g^−1^)	*E*_a_ (kJ mol^−1^)	TOF × 10^3^^b^ (s^−1^)
Cu/Z	267.3	0.16	2.51	0.43	23.9	24.7
Ca/Cu/Z	249.1	0.16	2.51	0.37	32.3	21.1
Ca/Zr_0.05_Cu/Z	242.1	0.16	2.58	0.54	26.9	16.1
Ca/Zr_0.10_Cu/Z	247.2	0.15	2.48	0.62	24.2	15.8
Ca/Zr_0.20_Cu/Z	248.8	0.15	2.50	0.52	26.4	17.1
Ca/Zr_0.40_Cu/Z	220.8	0.14	2.59	0.42	27.2	19.5
Zr_0.10_Cu/Z	279.1	0.16	2.41	0.71	23.2	15.7

a Determined by N_2_O titration method.

b Calculated using the eqn (4) at the reaction temperature of 140 °C.

The metallic copper surface area (*S*_Cu_) was measured with the N_2_O titration method and presented in Table 1. After Ca loading, the *S*_Cu_ of Cu/Z catalyst decreases from 0.43 to 0.37 m^2^ g^−1^, which is ascribed to the coverage of surface copper by CaO. With the addition of zirconium, the *S*_Cu_ of the catalysts increased remarkably, and a maximum of 0.62 m^2^ g^−1^ was obtained for the Ca/Zr_0.10_Cu/Z catalyst. This phenomenon indicates that the dispersion of copper can be promoted by the addition of an appropriate amount of zirconium. In comparison with Cu/Z catalyst, the reference catalyst Zr_0.10_Cu/Z possess a much larger *S*_Cu_ (0.71 m^2^ g^−1^), which further verifies the promotion of Zr for the dispersion of Cu. The reason for this is attributed to that the Zr doping inhibits the aggregation of CuO species on the surface of catalyst.

**Fig. 1:**
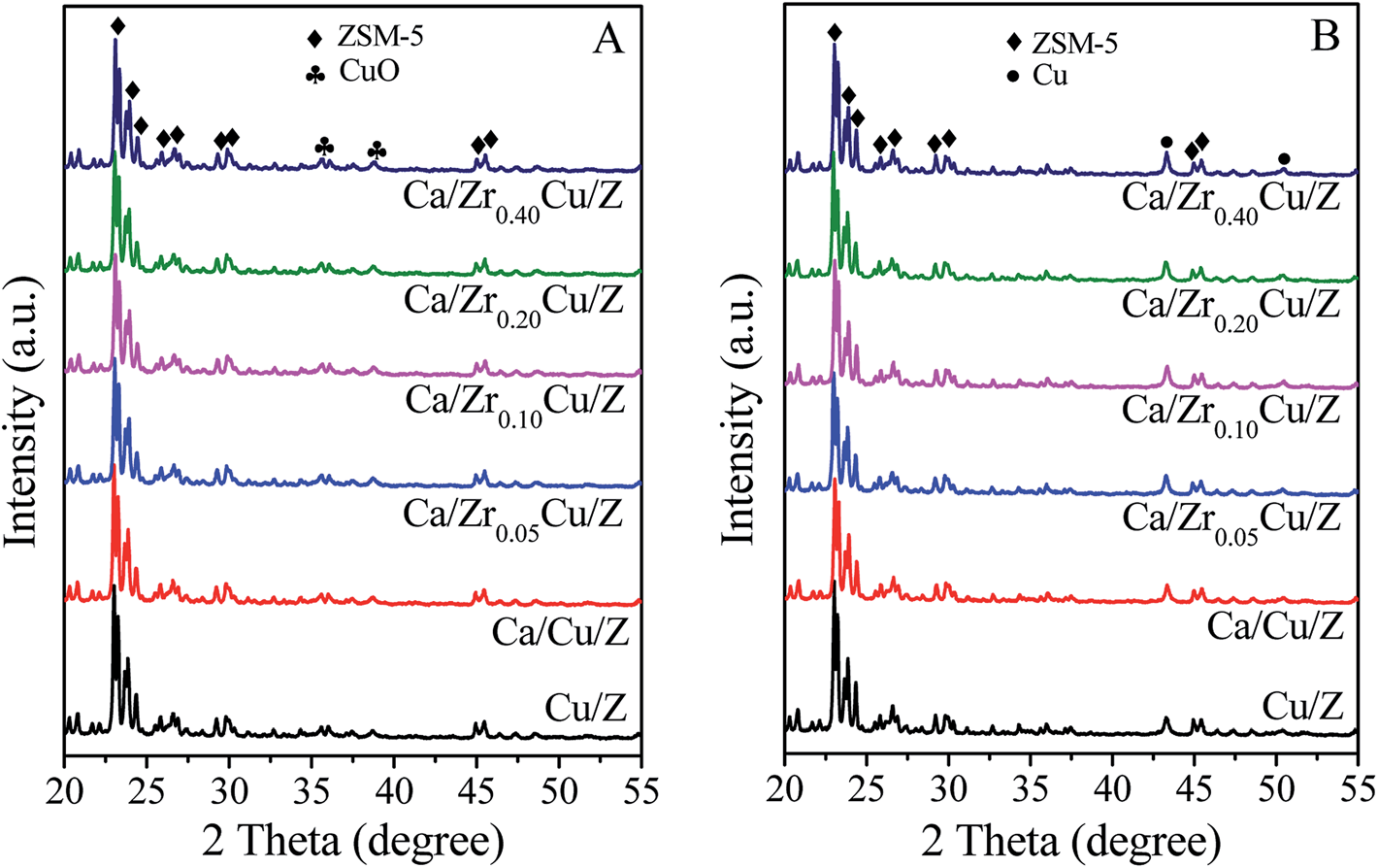
XRD patterns of Cu/Z and Ca/Zr_*x*_Cu/Z catalysts: (A) calcined catalysts and (B) reduced catalysts.

The XRD patterns of the Cu/Z and Ca/Zr_*x*_Cu/Z catalysts are shown in Fig. 1(A). All the samples exhibit the characteristic diffraction peaks of ZSM-5 (PDF #44-0003), which indicates that the zeolite structure remains unchanged after the impregnation of copper, calcium and zirconium. With increasing zirconium content, the intensity of diffraction peaks of ZSM-5 zeolite decreased slightly. The weak diffraction peaks at 35.5° and 38.7° correspond to the CuO (PDF #45-0937) suggesting the formation of crystalline CuO over Cu/Z and Ca/Zr_*x*_Cu/Z. For all the samples, no characteristic diffraction peak of zirconium oxide and calcium oxide was observed, and the result implies that the zirconium and calcium species exist in a low degree of crystallinity or in a high dispersion. Moreover, the XRD patterns of the Ca/Zr_*x*_Cu/Z catalysts reduced at 300 °C were obtained and presented in Fig. 1(B). The characteristic peaks of the cubic metallic Cu were observed at 43.5° and 50.4° for all the samples accompanied with the disappearance of characteristic peaks of CuO, and no diffraction peak of Cu^+^ was detected. These results suggested that CuO in the sample was transformed into Cu^0^ after the reduction.

### 3.2. NH_3_-TPD analysis

**Fig. 2:**
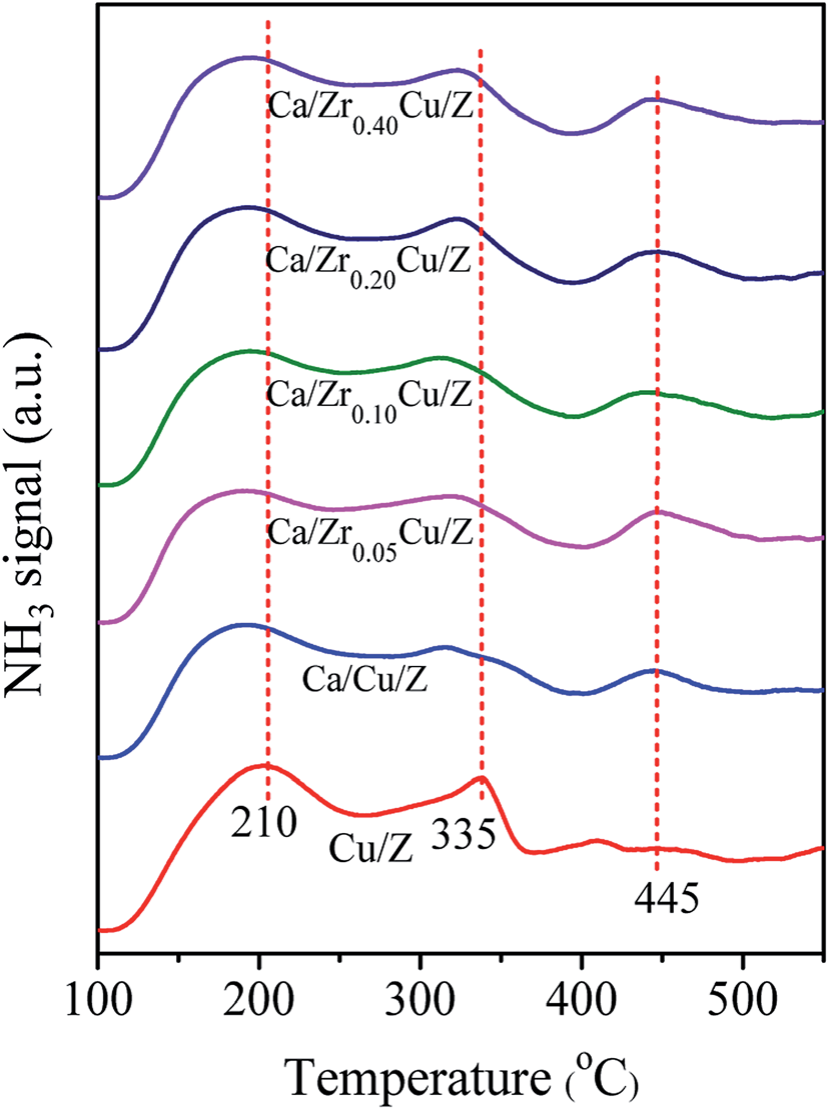
NH_3_-TPD profiles of Cu/Z and Ca/Zr_*x*_Cu/Z catalysts.

For the NH_3_-SCR reaction, it is important to determine the surface acidity of catalyst.^30^ The NH_3_ desorption profiles over Cu/Z and Ca/Zr_*x*_Cu/Z catalysts are shown in Fig. 2. Three desorption peaks were observed at 210, 335 and 445 °C for the Cu/Z catalyst. Here, the peaks located at 210 and 445 °C can be reasonably assigned to the weak acid sites and strong acid sites over the surface of HZSM-5, respectively.^21,31,32^ The peak located at 335 °C corresponds to the desorption of NH_3_ from the metal ion (Cu^2+^ in this case).^21,33–35^ After the Ca impregnation, the peak at 335 °C weakened indicating that the loading of calcium reduces the amount of Lewis acid sites. Compared with Ca/Cu/Z sample, no significant change in the NH_3_-desorption profiles can be discerned over the Ca/Zr_*x*_Cu/Z catalysts, and this result illustrates that the addition of zirconium exerts little influence on the acidity of Ca/Cu/Z catalyst.

### 3.3. H_2_-TPR analysis

**Fig. 3:**
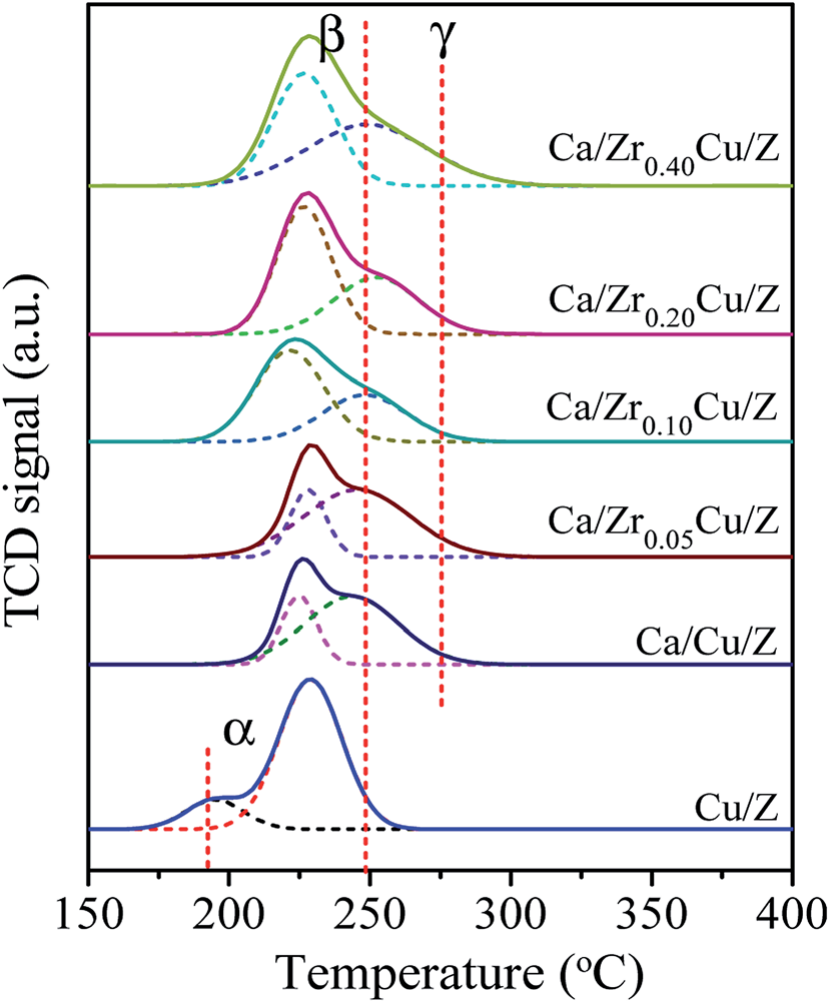
H_2_-TPR profiles of Cu/Z and Ca/Zr_*x*_Cu/Z catalysts.

The H_2_-TPR profiles of the Cu/Z and Ca/Zr_*x*_Cu/Z samples are presented in Fig. 3. There are two reduction peaks in the TPR profile of the Cu/Z catalyst appearing at about 195 °C and 230 °C, respectively. The low temperature peak (α peak) is related to the highly dispersed CuO on the surface of catalyst, while the high temperature reduction peak (β peak) can be assigned to the bulk CuO.^32^ After CaO loading, α peak disappeared because the highly dispersed copper species was covered by CaO. Moreover, β peak became broad and a shoulder appeared on the high-temperature side (denoted as γ peak). There is an interaction between copper species and CaO, which decreases the reducibility of CuO.^21^ Therefore, γ peak corresponds to the reduction of bulk-like CuO interacted with CaO strongly, and a similar result was reported by Liu et al.^36^ The positions and relative contributions of reduction peak (β and γ peak) of the Ca/Zr_*x*_Cu/Z catalysts are summarized in Table 2. With the introduction of Zr, a small shift of β peak towards the lower temperature can be observed suggesting a higher copper dispersion. Furthermore, the fraction of β peak in the TPR pattern increases first and then decreases with the increase in Zr loading, and a maximum was obtained over the sample of Ca/Zr_0.10_Cu/Z. These results indicate that the introduction of appropriate amount Zr increase the copper dispersion and weaken the interaction between the CuO and CaO.

**Table 2:** Temperature of reduction peaks and their contribution to the H_2_-TPR profiles of Ca/Zr_*x*_Cu/Z catalysts

Catalyst	Peak β		Peak γ	
	Temperature (°C)	Fraction (%)	Temperature (°C)	Fraction (%)
Ca/Cu/Z	225	27.8	244	72.2
Ca/Zr_0.05_Cu/Z	228	29.0	245	71.0
Ca/Zr_010_Cu/Z	221	61.2	248	38.8
Ca/Zr_0.20_Cu/Z	226	59.5	250	40.5
Ca/Zr_0.40_Cu/Z	226	48.4	249	51.6

Some researchers^37,38^ reported that there are two types divalent copper (Cu(ii)) in the zeolite supported copper catalysts. One is the common Cu(ii) existing in the CuO crystallite (denoted as cry-CuO); the other is the isolated Cu^2+^ (denoted as iso-Cu^2+^), which forms *via* the ion-exchange between Cu^2+^ and zeolite during the catalyst preparation process. The reduction of cry-CuO is a one-step reduction, *i.e.* CuO reduces to Cu^0^ directly, and the reduction generally occurs in the temperature range of 200–300 °C. Two steps are involved in the reduction of iso-Cu^2+^: first, the isolated Cu^2+^ reduces to isolated Cu^+^ (iso-Cu^+^) in the range of 200–300 °C; second, at a temperature above 330 °C, the reduction of iso-Cu^+^ to Cu^0^ happens. However, in this case, there are no peaks corresponding to the reduction of iso-Cu^2+^ and iso-Cu^+^. The reason is ascribed to that, with the wet impregnation method to prepare Cu/zeolite catalyst, the amount of iso-Cu^2+^ is too small to be detected by TPR technique. The XRD results of catalysts reduced at 300 °C also confirmed the absence of iso-Cu^+^.

### 3.4. XPS analysis

**Fig. 4:**
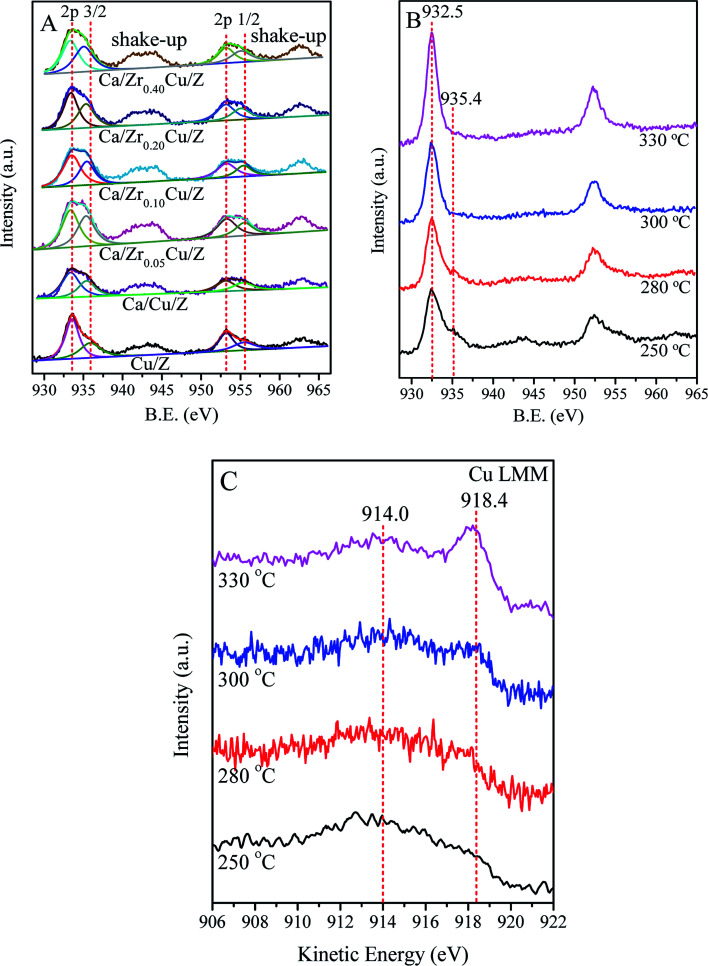
Cu 2p XPS spectra of Cu/Z and Ca/Zr_*x*_Cu/Z catalysts: (A) fresh catalysts calcined in air; (B) Ca/Zr_0.10_Cu/Z catalyst reduced in H_2_ under different temperature; and (C) Cu (LMM) AES of Ca/Zr_0.10_Cu/Z catalysts reduced in H_2_ under different temperature.

Fig. 4(A) shows the results of the Cu 2p of the calcined Cu/Z and Ca/Zr_*x*_Cu/Z samples. The binding energies (BE) of Cu 2p_3/2_ and Cu 2p_1/2_ appear at around 933.6 eV and 953.2 eV, respectively.^39,40^ The presence of shakeup satellite peaks at about 943.0 eV and 963.0 eV reveals that the divalent copper (Cu(ii)) is presented in Cu/Z and Ca/Zr_*x*_Cu/Z catalysts. The spectra of Cu 2p_3/2_ of all catalysts were deconvoluted into two peaks at around 933.0 eV and 935.4 eV. The former corresponds to the Cu(ii) in CuO crystallites (cry-CuO), as demonstrated by the XRD results; the latter can be assigned to the isolated Cu^2+^ (iso-Cu^2+^) species produced during the catalyst preparation process.^6,37^ As shown in Table 3, the peaks of Cu 2p_3/2_ on the Cu/Z catalyst slightly shift towards lower energy with Ca loading. The reason for this is that the Ca with a higher electron donating ability can drive electrons to Cu resulting in a lower BE of Cu species.^21^ Furthermore, the effect of the amount of zirconium on the BE values of Cu 2p_3/2_ is indiscernible.

**Table 3:** XPS results for Cu/Z and Ca/Zr_*x*_Cu/Z catalysts

Catalyst	Surface concentration (at%)			Cu 2p_3/2_ (B.E.)		Cu 2p_1/2_ (B.E.)		Cry-CuO/iso-Cu^2+^^a^
	Cu	Ca	Zr	CuO	Iso-Cu^2+^	CuO	Iso-Cu^2+^	
Cu/Z	1.22	—	—	933.6	935.9	953.2	955.5	1.72
Ca/Cu/Z	1.06	1.12	—	933.3	935.4	953.1	955.3	1.49
Ca/Zr_0.05_Cu/Z	1.90	1.19	0.24	933.3	935.3	953.1	955.3	1.40
Ca/Zr_0.10_Cu/Z	2.16	1.15	0.36	933.3	935.4	953.1	955.3	1.49
Ca/Zr_0.20_Cu/Z	1.75	1.19	0.48	933.3	935.3	953.1	955.3	1.46
Ca/Zr_0.40_Cu/Z	1.70	1.22	0.71	933.3	935.3	953.2	955.3	1.42

a The peak area ratio of CuO crystallites to isolated Cu^2+^ ions in XPS spectrum.

The surface compositions of the catalysts were also presented in Table 3. The amount of surface Cu atoms of the Cu/Z catalysts reduces from 1.22% to 1.06% with Ca loading. With the addition of Zr, the amount of surface Cu of Ca/Cu/Z catalysts increases remarkably, indicating that Zr enriches the copper species on the catalyst surface. The amount of Cu atom reached a maximum (2.16%) as the zirconium of 0.10 wt% was loaded. As for the amount of Ca atom on the surface of the catalysts, only a slight change can be found with the increase in Zr loading. It was well-documented that, for the NH_3_-SCR reaction, cry-CuO and iso-Cu^2+^ in Cu/zeolite were both the active sites.^6,37,41^ The ratios of cry-CuO to iso-Cu^2+^ were calculated from the corresponding peak areas in XPS spectra and presented in Table 3. The result showed that cry-CuO was predominant on the surface of all catalysts, but with the addition calcium, the ratio of cry-CuO to iso-Cu^2+^ decreased from 1.72 to 1.49. After the addition of zirconium in the Ca/Cu/Z catalyst, the ratio of cry-CuO to iso-Cu^2+^ is almost a constant.

In order to get further insight into the change in chemical state of copper element on the surface of catalyst, *in situ* XPS for the catalyst reduced at different temperature was carried out in this case. Fig. 4(B) shows the results of the Cu 2p of Ca/Zr_0.10_Cu/Z catalyst reduced at 250, 280, 300 and 330 °C. With the increase in the reduction temperature, the shakeup feature of the Ca/Zr_0.10_Cu/Z catalyst weakened gradually. As the temperature reaches 300 °C, the shakeup peaks disappeared completely, and the characteristic feature of the iso-Cu^2+^ species (*ca.* 935.4 eV) vanished. These results indicated that both the cry-CuO and iso-Cu^2+^ were reduced completely at 300 °C. However, the Cu^0^ and Cu^+^ species cannot be differentiated because the Cu 2p_3/2_ BE values of Cu^0^ and Cu^+^ are nearly identical (*ca.* 932.5 eV). Fortunately, the LMM Auger spectra of Cu^0^ (∼918.8 eV) and Cu^+^ (existed as Cu_2_O, ∼916.8 eV) are separated by approximately 2.0 eV, and they can be used to distinguish the valence state of Cu. In this work, the Auger electron spectroscopy of the Ca/Zr_0.10_Cu/Z catalyst at the reduction temperature of 250, 280, 300 and 330 °C were also collected and presented in Fig. 4(C). At 250 °C, the Cu (LMM) Auger peaks centered at around 914.0 eV can be observed suggesting the existence of isolated Cu^+^ (iso-Cu^+^).^42^ It is worth to mention that, the Cu Auger is peculiarly sensitive to chemical state,^42–44^ and the Auger spectra of iso-Cu^+^ is different from that of Cu^+^ in Cu_2_O. In this case, the Auger peak appears at around 914.0 eV rather than 916.8 eV. With the increase in the reduction temperature, the Auger peaks of Cu^+^ became weaker accompanied with an increase in the peak intensity of 918.4 eV (Cu^0^). When the reduction temperature is up to 330 °C, Cu^+^ was reduced almost completely to Cu^0^.

### 3.5. SCR activity and kinetic measurements

**Fig. 5:**
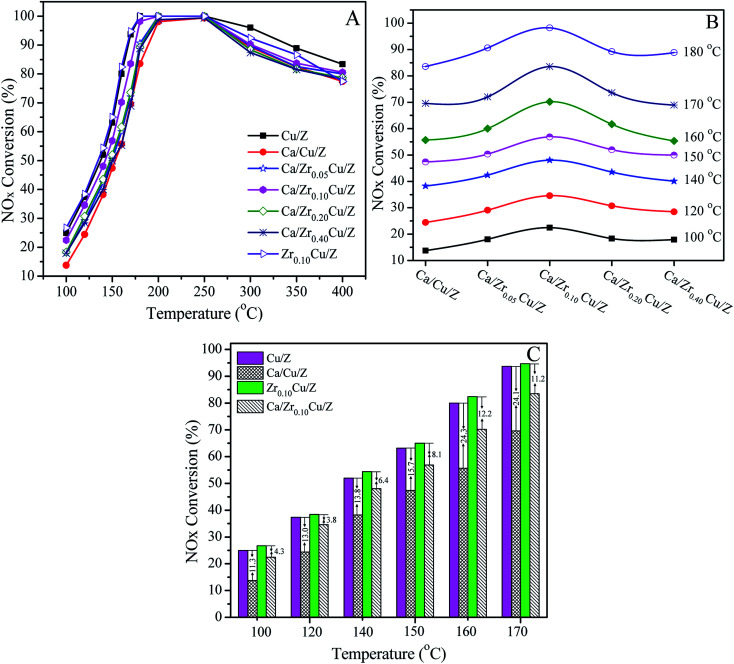
Catalytic activity of Cu/Z and Ca/Zr_*x*_Cu/Z catalysts for the NH_3_-SCR reaction: (A) NO_*x*_ conversion as a function of the reaction temperature, (B) NO_*x*_ conversion as a function of Zr loading, and (C) NO_*x*_ conversion over Cu/Z and Zr_0.10_Cu/Z with/without calcium loading. Reaction conditions: [NO] = [NH_3_] = 500 ppm, [O_2_] = 5%, N_2_ balanced, flow rate = 200 ml min^−1^, GHSV = 80 000 h^−1^.

The catalytic activities of Cu/Z and Ca/Zr_*x*_Cu/Z catalysts for the NH_3_-SCR are presented in Fig. 5(A). All the investigated catalyst exhibits a typical behavior of the SCR of NO_*x*_. First, the NO_*x*_ conversion increases with increasing the reaction temperature, and after reaching a maximum at the intermediate temperature, the NO_*x*_ conversion begins to decrease since the oxidation of ammonia with O_2_ become prevalent at a high temperature.^45,46^ As shown in Fig. 5(A), after the impregnation of Ca, the catalytic activity of the Cu/Z catalyst declined over the whole temperature range due to the toxic effect of CaO.^21^ With the introduction of zirconium, the activity of Ca/Cu/Z improved remarkably, particularly in the low-temperature region. Fig. 5(B) shows the variation of NO_*x*_ conversion with the change in the content of zirconium in the temperature range of 100–180 °C. The activity of Ca/Zr_*x*_Cu/Z catalyst takes on a volcano-shape variation, and the highest catalytic activity is obtained over Ca/Zr_0.10_Cu/Z catalyst. To gain further insight into the enhanced calcium poisoning resistance of the Zr-modified Cu/Z catalyst, NO_*x*_ conversion over the Cu/Z and Zr_0.10_/Cu/Z catalysts with/without the calcium loading in the temperature range of 100–170 °C are shown in Fig. 5(C). After the calcium impregnation, NO_*x*_ conversion over Cu/Z catalyst decreased by 11.3–24.3%, while that over Zr_0.10_/Cu/Z only decreased by 3.8–12.2%. Clearly, the resistance to calcium poisoning over the Zr_0.10_/Cu/Z catalyst improved remarkably.

As reported in our previous work,^21^ the poisoning effect of calcium on Cu/ZSM-5 is related to the decrease in the surface Cu atoms, the decline in the reducibility of CuO and the loss of the surface acid sites. In this case, as demonstrated by the results of *S*_Cu_ and XPS, the addition of zirconium leads to an increase in the copper dispersion and the surface concentration of copper atoms. As shown in the part of H_2_-TPR, with the addition of zirconium, the interaction between CuO and CaO decreases, and the reducibility of CuO is improved. Moreover, as illustrated in the Fig. 2, there is no significant change in the surface acid sites. Therefore, in this study, the enhanced resistance to Ca poisoning results from the improvement of the Cu dispersion and the reducibility of CuO.

**Fig. 6:**
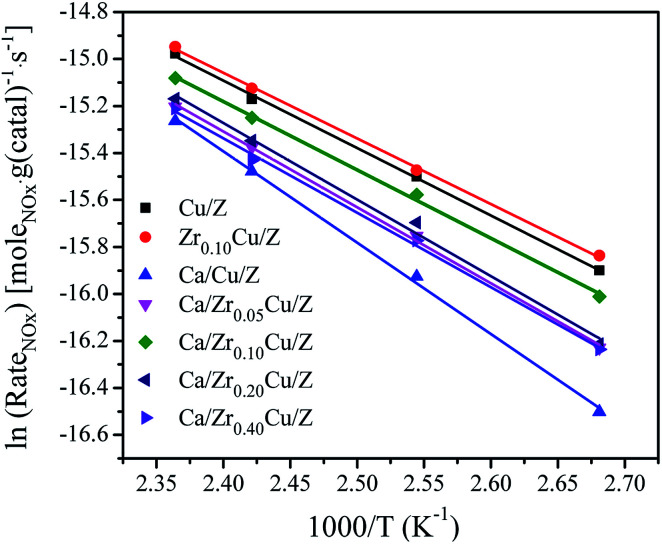
Arrhenius plots of the SCR reaction rates per gram of catalyst at 100–150 °C over Cu/Z and Ca/Zr_*x*_Cu/Z catalysts. Reaction conditions: [NO] = [NH_3_] = 500 ppm, [O_2_] = 5%, N_2_ balanced, flow rate = 200 ml min^−1^, GHSV = 80 000 h^−1^.

The NH_3_-SCR reaction rates per gram of catalyst over Cu/Z and Ca/Zr_*x*_Cu/Z catalysts were calculated by eqn (3) in the temperature range 100–150 °C. In such a temperature range, the conversions of NO_*x*_ are low, and the reaction is far from thermodynamic equilibrium. Arrhenius plots of the SCR reaction rates over Cu/Z and Ca/Zr_*x*_Cu/Z catalysts are shown in Fig. 6. According to the linear relationship between the reaction rate and the reciprocal of temperature, the apparent activation energy (*E*_a_) was determined. As shown in Table 1, the *E*_a_ for NH_3_-SCR reaction of Cu/Z catalyst is similar to that of Zr_0.10_Cu/Z catalyst. The apparent activation energy increases over Cu/Z catalyst after Ca loading. While with the addition of zirconium, the apparent activation energy of Ca/Cu/Z catalyst decreases, and Ca/Zr_0.10_Cu/Z catalyst possessed the minimum value of apparent activation energy in all Ca-impregnating catalysts. Evidently, the variation trend of the apparent activation energy with the increase in the amount of Zr is agreement with that of NO_*x*_ conversion.

Turnover frequencies (TOF) of NH_3_-SCR reaction at 140 °C, which represents the number of NO_*x*_ molecules converted per metallic copper atom per second, was calculated by eqn (4) for the investigated catalysts, and the results are also presented in Table 1. The values of TOF varied in the range of 15.7–24.7 × 10^−3^ s^−1^. After the Ca impregnation, the value of TOF over the Cu/Z catalyst decreases. With the addition of zirconium, the TOF of Ca/Zr_*x*_Cu/Z catalysts decreases further, and a minimum is observed on the Ca/Zr_0.10_Cu/Z catalyst. The result of TOF reveals that the introduction of zirconium improves the catalytic activity *via* increasing the amount of Cu active sites not the activity per Cu-site. Actually, instead of increasing, the activity per Cu-site decreases. Moreover, the TOF varies with the change in the dispersion of Cu suggests that the SCR reaction over the Ca/Zr_*x*_Cu/Z catalyst is a structurally sensitive reaction.

## 4. Conclusion

With the addition of zirconium, an enhanced calcium poisoning resistance of Cu/ZSM-5 catalyst for the SCR of NO was obtained. The introduction of zirconium gives rise to an increase in *S*_Cu_ and the surface concentration of copper atoms, which is the main reason responsible for the improvement of catalytic activity. The introduction of zirconium decreases the interaction between the CuO and CaO and further promotes the reduction of CuO. Moreover, there is no significant change in the acidity of Ca/Cu/Z after the addition of zirconium. The optimum addition amount of zirconium is 0.10%, and the highest catalytic activity was obtained over the corresponding catalyst.

## Conflicts of interest

There are no conflicts to declare.

## Supplementary Material
